# Nano‐Based Sodium Stibogluconate for Treatment of Cutaneous Leishmaniasis Caused by *Leishmania major* in Male Mice

**DOI:** 10.1155/japr/5461643

**Published:** 2026-10-22

**Authors:** Zahra A. Alamri, Noha T. Zelai, Amaal H. Hassan

**Affiliations:** ^1^ Department of Biological Sciences Faculty of Science King Abdulaziz University Jeddah Saudi Arabia kau.edu.sa; ^2^ Department of Biology Faculty of Science King Khalid University Abha Saudi Arabia kku.edu.sa

**Keywords:** chitosan, cutaneous leishmaniasis, mannose, nanoparticles, nanosystems, sodium stibogluconate

## Abstract

The World Health Organization (WHO) stated that 700,000 to 1 million people around the world contract Leishmaniasis annually. Saudi Arabia has been recorded as one of the six countries with the highest infection rate (up to 90%). In this study, novel nanomedicine formulations of sodium stibogluconate (SSG), including chitosan nanoparticles and mannose‐decorated chitosan nanoparticles, were prepared for the treatment of cutaneous leishmaniasis caused by *Leishmania major* in male mice. The in vivo pharmacokinetics of these formulations was tracked at several time points. By the end of the administered treatment period, the effectiveness of the treatment was evaluated using enzyme‐linked immunosorbent assay and quantitative real‐time PCR. The results showed that the nanoformulations of SSG (SSG CS NPs and SSG M CS NPs) maintained plasma drug concentrations approximately three times higher than those of free SSG after 72 h, indicating extended drug release and prolonged bioavailability. IgG levels were the lowest in the group treated with SSG M CS NPs, followed by those in SSG CS NPs and then the free SSG group. qPCR results also showed higher *C* *t* values for the drug‐enhanced nanoformulations (SSG CS NPs and SSG M CS NPs), indicating reduced parasitic DNA and increased treatment efficacy. These findings demonstrate that nanocarriers, especially mannose‐modified nanoparticles, may be associated with the improved drug delivery properties, as supported by the in vitro release profile and pharmacokinetic results.

## 1. Introduction

Leishmaniasis, a chronic and neglected health problem prevalent worldwide, is a parasitic ailment arising from the flagellated protozoan *Leishmania* [1]. *Leishmania* parasites inhabit macrophages of the mammalian host, which actively mount immune responses against infection, whereas intracellular amastigotes employ immune evasion strategies to persist and replicate within these cells [2]. Activated macrophages are involved in parasite management by producing proinflammatory cytokines, nitrogen intermediates, and reactive oxygen [3]. However, *Leishmania* amastigotes interfere with signaling pathways and inhibit antimicrobial effector functions to persist within these cells [4, 5] Leishmaniasis is transmitted by female phlebotomine sandflies, including 30 species of *Lutzomyia* in North America and South America and more than 45 species of *Phlebotomus* in Asia, Europe, and Africa [6]. Canine and rodent species, as reservoir hosts, play a pivotal role in maintaining the *Leishmania* life cycle [7]. Species of the genus *Leishmania* cause various forms of leishmaniasis. They can cause cutaneous leishmaniasis, which is characterized by localized skin lesions typically beginning as papules or nodules at the site of the sandfly bite and may progress to ulcerative lesions. Although often self‐limiting, cutaneous leishmaniasis can result in chronic ulcers, secondary infections, and permanent scarring, leading to significant physical and psychosocial morbidity [8]. They may cause diffuse skin pustules, known as mucocutaneous leishmaniasis, with severe abnormalities at the site of infection. They can also cause a fatal disease called visceral leishmaniasis, which affects internal organs [9]. Notably, the World Health Organization considers leishmaniasis, in its various forms, an epidemic disease that is difficult to eradicate [10].

The first‐choice treatment for cutaneous leishmaniasis is sodium stibogluconate (SSG) [11]. This pentavalent antimony has been the standard treatment for all forms of leishmaniasis for over 80 years, with studies confirming a cure rate of at least 80% [12]. SSG fragments the DNA at the amastigote stage, leading to *Leishmania* apoptosis. It also inhibits DNA topoisomerase in the parasite, which prevents the replication and transcription of the parasite′s DNA [13, 14]. SSG was administered intravenously or intramuscularly at a dose of 15–20 mg/kg body weight daily for 21–28 days [15]. Intramuscular injections are often associated with local pain and side effects such as vomiting, nausea, muscle and joint pain, diarrhea, rash, hepatotoxicity, and cardiotoxicity [14].

Intracellular amastigotes in a definitive host pose a challenge for drug delivery to macrophages. The drug must cross several biological barriers to reach and eliminate amastigotes inside the parasitophorous vacuoles in macrophages [16]. The use of nanotechnology to deliver medicines for the treatment of various diseases has made it possible to overcome the obstacles faced by traditional medicine [17]. The use of nanosystems to deliver antileishmanial drugs has several advantages. Nanodelivery systems can reduce the toxicity of conventional drugs, efficiently target infected cells, and increase drug bioavailability in the body. These benefits result in reduced drug doses, prevention of drug resistance [18], and protection of drugs from immediate exposure to metabolic or enzymatic degradation in the gastrointestinal tract, liver, and circulatory system [19].

Previous studies have investigated nano‐based delivery systems to enhance the therapeutic performance of SSG against leishmaniasis. Various nanocarriers, including niosomal [20] and liposomal [21], have been reported to improve the physicochemical stability of SSG and promote its delivery to the infected macrophages. These nanoformulations demonstrate enhanced antileishmanial efficacy in experimental models, as evidenced by significant reductions in parasite burden, improved lesion healing, and prolonged drug availability at the site of infection. Moreover, nanoencapsulation of SSG has been shown to facilitate greater intracellular drug exposure within macrophages, thereby enhancing the interaction between the drug and intracellular amastigotes. Collectively, these findings from the literature highlight the potential of nanocarriers delivery platforms to optimize SSG distribution and therapeutic outcomes in leishmaniasis [22] (Table S1). This study is aimed at preparing and characterizing novel chitosan and mannose‐decorated chitosan nanoparticles (M CS NPs–CS NPs) to improve the delivery of SSG and treat *Leishmania major* in vivo. These formulations were used to treat cutaneous leishmaniasis‐infected mice. The in vitro release profile and the pharmacokinetics of all drugs in blood plasma were monitored at several different time points. The efficacy of these novel formulations was evaluated using molecular and immunological methods.

Chitosan has emerged as one of the most widely investigated natural polymers for nanoparticle‐based drug delivery owing to its excellent biocompatibility, biodegradability, low toxicity, and cationic nature [23]. The protonated amino groups of chitosan confer a positive surface charge that enhances interactions with negatively charged biological membranes and provides reactive sites for chemical conjugation with various targeting ligands. These physicochemical properties facilitate nanoparticle functionalization while maintaining formulation stability and drug encapsulation efficiency (EE) [24]. Among the available targeting ligands, mannose has attracted considerable interest because it can be readily conjugated to chitosan through its reactive amino groups, enabling surface modification of nanoparticles. Mannose‐functionalized nanoparticles have been extensively investigated as a targeted delivery strategy because of the expression of mannose receptors (CD206) on macrophages, which are the primary host cells harboring *Leishmania* amastigotes [25]. Previous studies have reported that mannose receptor‐mediated endocytosis may enhance the interaction and cellular uptake of mannose‐functionalized nanoparticles by macrophages, thereby promoting intracellular drug delivery [26, 27]. Based on these findings, mannose surface functionalization was incorporated in the present study as a rational design strategy to improve the interaction of sodium SSG‐loaded chitosan nanoparticles (CS NPs) with macrophages while recognizing that receptor‐mediated targeting was not directly evaluated in the present work.

## 2. Materials and Methods

### 2.1. Animals

Male BALB/c mice, with an average age of 2–3 months and weights ranging from 25–30 g, were randomly divided into cages (nine per cage). Male mice were used consistently across all experimental groups to maintain a uniform experimental population and avoid introducing sex as an additional biological variable into the study design. All mice had access to water and food ad libitum. Mice were maintained under normal environmental conditions, with a constant condition of 24°C in a 12‐h light/12‐h dark photoperiod. This study was ethically approved by the Assiut Veterinary Medicine Research Ethics Committee (Reference No: 0354).

### 2.2. *Leishmania* Parasite

*Leishmania major* promastigotes were cultured in RPMI‐1640 medium with L‐glutamine, 10% fetal bovine serum, and penicillin streptomycin. Parasite cultures were incubated at 26°C in a 5% CO_2_. Promastigotes were counted using a hemocytometer slide and diluted in phosphate buffered saline. Each mouse was injected subcutaneously in the sacral region with 1 × 10^7^ promastigotes for experimental infection [28].

### 2.3. Preparation of Drugs

#### 2.3.1. Free SSG and Vehicle

Free SSG was prepared by placing 4.0 g in a sterile beaker with 30 mL of distilled water (dH_2_O) and gently stirring until the SSG was fully dissolved. The volume was made up to 40 mL with dH_2_O. A sterile syringe filter (0.22 *μ*m) was used to remove any microcontaminants and sterilize the solution before filling it into sterile glass vials and storing it at 2°C–8°C [29]. The blank vehicle contained only hot dH_2_O.

#### 2.3.2. Blank CS NPs

According to the ionotropic gelation process, CS NPs were prepared in accordance with the previously described protocol. [30, 31]. An aqueous solution of tripolyphosphate (TPP) was added to the chitosan solution to obtain blank CS NPs. Then, 1.0 g chitosan was solubilized in 0.2 L 1% CH_3_COOH. The solution was swirled for 6 h until homogeneous. Next, 150 mL of TPP was added in drops. The formation of CSNPs was indicated when the solution became clear. Then, the mixture was centrifuged thrice with dH_2_O at 15,000 rpm for 30 min (Hermle Z32 HK, Germany).

#### 2.3.3. SSG Encapsulated CS NPs

SSG was dissolved in a chitosan solution following the methods described previously [32, 33], with slight modifications. Briefly, 0.1 g of SSG powder was dissolved in 100 mL of hot dH_2_O and added dropwise into chitosan solution (0.5% w/v in 1% acetic acid and containing 0.5% w/v Tween 80) in weight ratio 15% w/w. Thereafter, TTP was gradually added to the solution and stirred for 20 min. The mixture was then centrifuged at 4°C for 30 min at 15,000 rpm to separate the SSG‐loaded chitosan nanoparticles (SSG CS NPs).

#### 2.3.4. Mannoslayted‐CS NPs

Mannosylated SSG CS nanoparticles (SSG M CS NPs) were prepared as described previously [34]. Briefly, 2 mL of mannose solution was prepared by adding 10 mg of mannose to water at pH 4. This solution was then added to the previously prepared solution (SSG CS NPs) and stirred gently for 48 h. Under acidic conditions, the mannose surface functionalization of SSG CS NPs was performed according to the previously reported method described by Vieira et al. [35]. Impurities and unencapsulated mannose particles were removed by applying the previous solution (SSG M CSNPs) to a dialysis membrane (12 kDa; SERVA, Germany) for 30 min with double‐dH_2_O in the receptor compartment. Blank CS NPs were formulated similarly [36].

### 2.4. Characterization of the Synthesized Nanoparticles

#### 2.4.1. Size and Shape

In this work, transmission electron microscopy (TEM) analysis was performed using a high‐resolution transmission electron microscope (JEOL JEM‐2100) operated at an accelerating voltage of 200 kV. The samples were prepared by dispensing a drop of the nanoparticle colloid onto a carbon‐supported copper grid and allowing it to dry under ambient laboratory conditions. After which, the particle size distribution and average size were assessed using an image analysis software package.

#### 2.4.2. Determination of EE

The amount of encapsulated SSG in the NP was determined as follows: The antimony content in a known concentration (1 mg/mL) and in the supernatant samples was measured using inductively coupled plasma mass spectrometry (ICP‐MS; Avio 500 ICP, PerkinElmer) after high pressure microwave digestion. After separating the NPs from the reaction mixture, the response of the supernatant was measured, and the concentration of the standard sample was estimated. The EE was calculated according to the following equation:

Entrapment efficiency=Initial concentration−Free concentrationInitial concentration×100.

#### 2.4.3. Fourier Transform Infrared (FTIR) Spectroscopy

FTIR spectra with attenuated total reflectance were obtained using an FT‐IR Vertex 70 RAM II, Bruker Spectrometer. The spectra were recorded in the range of 4000–400 cm^−1^.

#### 2.4.4. Charge Density

A Zetasizer ZS90 (Malvern Instruments, Malvern, United Kingdom) was used to determine the zeta potential by suspending nanoparticles in deionized water.

#### 2.4.5. In Vitro Release Study

One gram of SSG CS NPs was PBS‐solubilized (5 mL) at physiological pH. After the suspension was placed in a dialysis bag (MWCO 10:12 KDa), the bag was immersed in 50 mL of PBS solution at 37°C. To determine the SSG concentration at time *t* (*C*_i_), the UV‐vis absorbance of the released SSG solution was recorded at a wavelength of 260 nm. The cumulative percentage of SSG released was calculated using the following equation:

Cumulative release=V0C0+V∑Ci−C0m×100,

where *V*_0_ is the total volume of the release solution (55.0 mL), *V* is the volume per sample (3.0 mL), and *m* is the initial loading amount of SSG in the nanocontainers.

### 2.5. Experimental Design

Each group contained nine mice (*n* = 9). The control groups include five groups: The first control group (negative control group) included mice that were not exposed to infection or treatment. The second control group (positive control group) included mice that were exposed to the infection but not treated. The third control group (SSG vehicle control) included mice infected with *Leishmania* and treated with water (the solvent of SSG) for 15 days. The fourth control group (CS NPs vehicle control) included mice infected with *Leishmania* and treated with a plain chitosan nanocarrier for 15 days. The fifth control group (M CS NPs vehicle control) included mice infected with *Leishmania* and treated with plain nanocarriers decorated with mannose for 15 days.

SSG‐treated mice were divided into three groups: The first group included mice infected with *Leishmania* and then treated with 20 mg/kg SSG solution for 15 days [37, 38]. The second group included mice infected with *Leishmania* and then treated with 20 mg/kg of SSG CS NPs for 15 days. The third group included mice infected with *Leishmania* and then treated with 20 mg/kg of SSG M CS NPS for 15 days (Figure 1).

**Figure 1:**
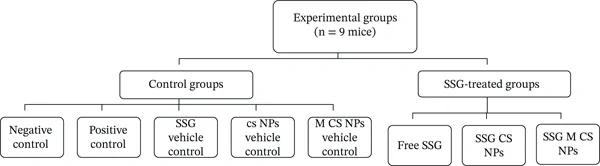
Schematic diagram of the experimental design.

### 2.6. Pharmacokinetics of SSG

The pharmacokinetics of SSG was monitored in the plasma of BALB/c mice at six time points (0, 1, 8, 24, 48, and 72 h). Blood samples ranging from 100 to 150 *μ*L were collected from the facial vein of the mice and immediately transferred into heparinized microtubes to prevent clotting, followed by 10‐min spinning at 4°C and 3000 rpm to separate the plasma. After plasma separation, the samples were stored at −80°C until use.

When used, the dithizone reagent was added to the plasma samples to form a color complex, which was measured at a wavelength of 530 nm. Absorbance readings were obtained with a Shimadzu UV‐1800 UV–Visible spectrophotometer (Japan). The results were recorded as shown in Table S2 and are presented graphically [39, 40].

### 2.7. Evaluation of Treatment Effectiveness

The study endpoint was defined at the completion of the 15‐day treatment course. After which, blood samples were collected from mice in all experimental groups under sterile conditions. The persistence of *Leishmania* after treatment was assessed by quantifying anti‐*Leishmania* antibodies using enzyme‐linked immunosorbent assay (ELISA) and parasite DNA using quantitative polymerase chain reaction (qPCR).

#### 2.7.1. ELISA

The ELISA method was performed as described by Sarkari et al. [41] with minor modifications. In this assay, soluble *Leishmania major* antigen (SLA) was prepared as previously described [42]. SLA was dissolved at a concentration of 10 *μ*g/mL in phosphate‐buffered saline. ELISA plates were coated with the antigen and left overnight at 4°C. The plates were then washed five times with saline containing Tween 20 (pH 7.4) to remove the excess antigen. Next was PBS addition and 1‐h incubation under laboratory conditions. Mouse serum was diluted (1:100) in PBS solution, with 100 *μ*L of serum put in every well, followed by 2‐h incubation at room temperature, rinsing of the plates three to five times using PBS‐Tween solution, addition of 100 *μ*L of IgG‐horse radish peroxidase, and 1‐h incubation at 37°C. The TMB substrate solution was added to the wells and incubated in the dark for 10–15 min. A stop solution (2 N H_2_SO_4_) was added to stop the reaction, and a microplate reader (Bio‐Rad Laboratories, United States) was employed to determine the absorbance at 450 nm (Table S3).

#### 2.7.2. Real‐Time qPCR Assay

Blood samples were collected from the tail veins of the study mice. DNA was extracted from the samples using the Qiagen DNeasy Blood DNA Extraction Kit according to the manufacturer′s instructions. Based on the study by Mary et al. [43], specific primers were designed to target the kDNA minicircle of *Leishmania major.*

The forward primer sequence was 5 ^′^‐CTTTTCTGGTCCTCCGGGTAGG‐3 ^′^, whereas the reverse primer sequence was 5 ^′^‐CCACCCGGCCCTATTTTACACCAA‐3 ^′^. The amplicon size was 120 bp long.

The following thermal reaction program was used: prior 10‐min denaturation at 95°C, 40, 15‐s denaturation cycles at 95°C, 30‐s annealing at 60°C, 30‐s extension at 72°C, and melting curve from 60°C to 95°C. A standard curve was drawn using samples with known concentrations of parasite DNA. The results were analyzed, and the parasite burden represented by the *C* *t* value was calculated (Table S4).

### 2.8. Statistics

The results were subjected to analysis using IBM SPSS Statistics 25. Results are shown as mean ± standard deviation. A one‐way ANOVA was conducted to evaluate statistical differences across the groups, followed by Tukey′s multiple comparison test. Results were considered statistically significant at a *p* value < 0.05.

## 3. Results

### 3.1. Characterization of the Synthesized Nanoparticles

#### 3.1.1. Size and Shape

The size and shape measurements of SSG CS NPs obtained using TEM showed that the average particle size was 32 ± 2 nm, and the particles exhibited a regular spherical structure (Figure 2).

**Figure 2:**
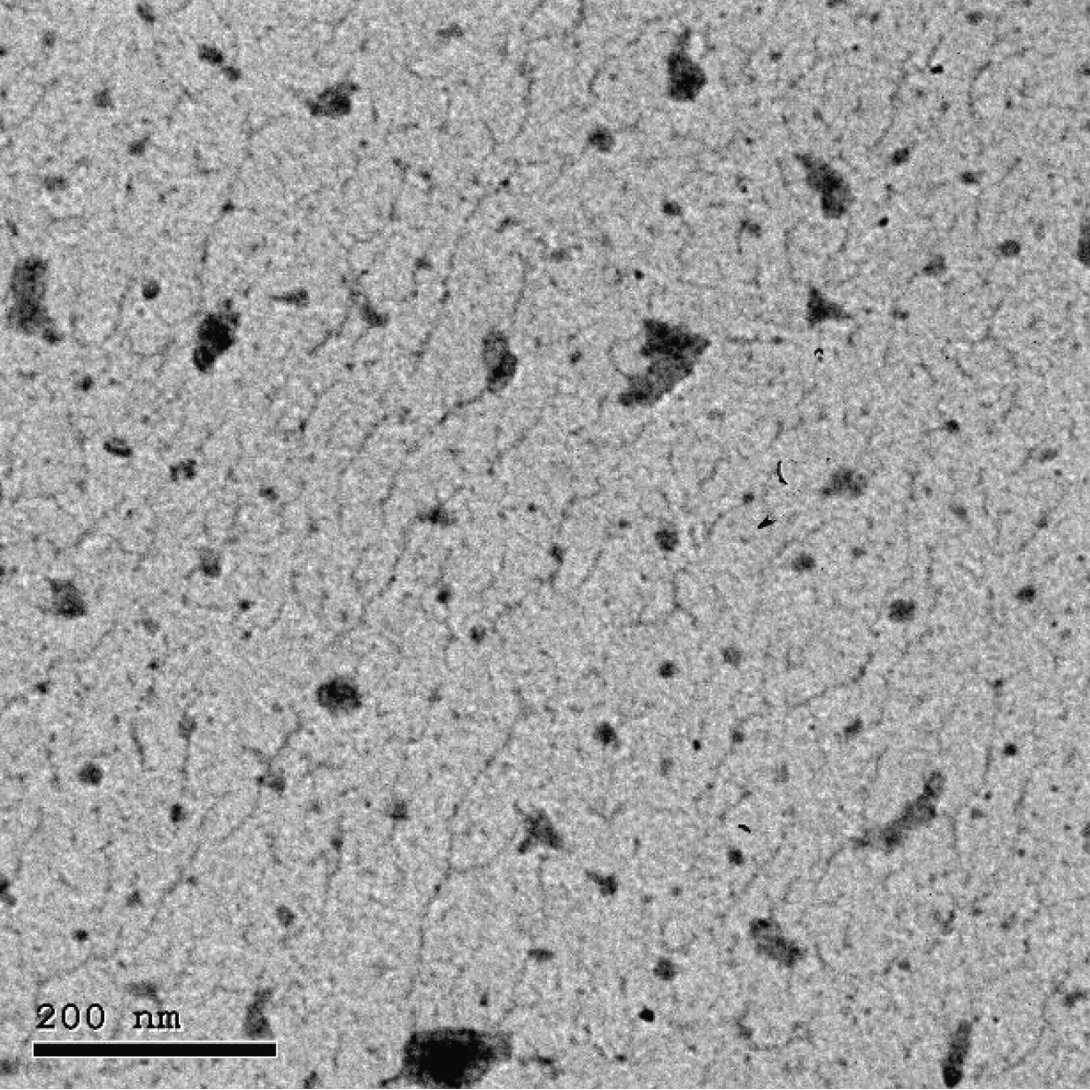
TEM image of prepared SSG CS NPs.

#### 3.1.2. Determination of EE

The loading efficiency of SSG, measured using inductively coupled plasma mass spectrometry (ICP‐MS), was approximately 65.31%.

#### 3.1.3. FTIR Spectroscopy

In CS NPs (dark blue curve), the broad peak located at 3360 cm^−1^ is attributed to the N–H stretching vibration, overlapping with the characteristic O–H stretching vibration. The –NH bending vibration peak appeared at 1635 cm^−1^ (Figure 3).

**Figure 3:**
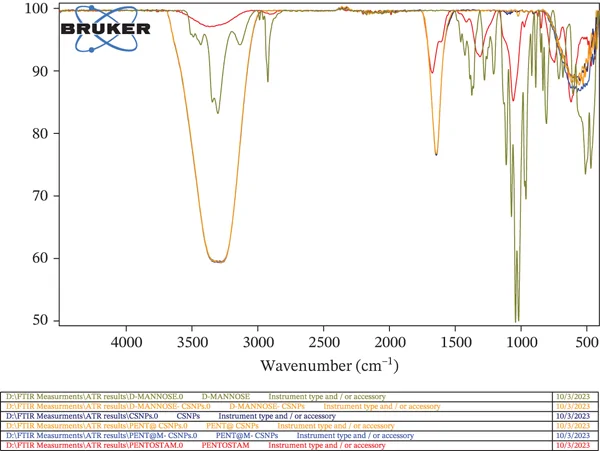
FTIR spectra of the prepared materials.

The FTIR spectrum of D‐mannose (green curve) revealed free –OH groups belonging to the asymmetric carbon atoms of the propylic bridge and oligomer chain terminus at approximately 3420 cm^−1^. The peaks at 3292 and 3127 cm^−1^ corresponded to the C–H stretching groups of the aromatic protective group from the sugar skeleton, whereas the CH_2_ from the furanose sugar rings displayed bands at 2978–2926 cm^−1^. Peaks at 1380 and 1035 cm^−1^ were due to the C–O–C asymmetric frequencies.

In the FTIR spectrum of the chitosan–mannose conjugate (orange curve), two characteristic peaks were observed at 1560 and 1456 cm^−1^, corresponding to the bending vibration of secondary amine (N–H) and the stretching of C=N, respectively. The appearance of these two peaks represents qualitative spectral features consistent with chemical modification of chitosan in the presence of mannose.

The main peaks of the vehicle (chitosan or mannose‐conjugated chitosan) did not change after the addition of SSG as no new peaks appeared, indicating the absence of new chemical bond formation (Figure 3).

#### 3.1.4. Charge Density

The surface charges of the nanoparticles were evaluated by measuring their zeta potentials (Table 1). SSG M CS NPs displayed a high zeta potential of approximately 28.8 ± 0.55 mV (Table 1).

**Table 1:** Zeta potential measurements of the prepared nanoparticles.

No.	Material	Zeta potential
1	SSG	−4.44 ± 0.41
2	CS NPs	40.7 ± 1.52
3	SSG CS NPs	31.3 ± 0.97
4	Mannose	−8.54 ± 0.814
5	M CS NPs	31.6 ± 0.95
6	SSG M CS NPs	28.8 ± 0.55

#### 3.1.5. In Vitro Release Study

The results of in vitro release study (Figure 4) indicated that the release of free SSG reached 90% after 350 h, whereas the release of SSG CS NPs reached 80% after 350 h (Figure 4). The mannose‐modified formulation was not included in the in vitro release experiment, as mannose functionalization primarily affects surface properties and cellular interactions rather than the diffusion‐controlled drug release behavior of the CS NP system.

**Figure 4:**
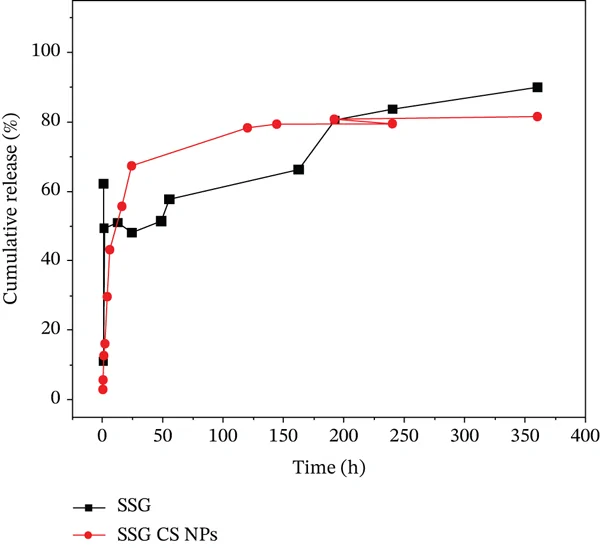
Representative in vitro cumulative release profiles of SSG and SSG CS NPs obtained using the dialysis bag method in PBS (pH 7.4) at 37°C. SSG, free sodium stibogluconate; SSG CS NPs, sodium stibogluconate chitosan nanoparticles.

#### 3.1.6. Pharmacokinetics of SSG

Figure 5 shows the pharmacokinetic results of SSG in the plasma of BALB/c mice across the three SSG treatment groups.

**Figure 5:**
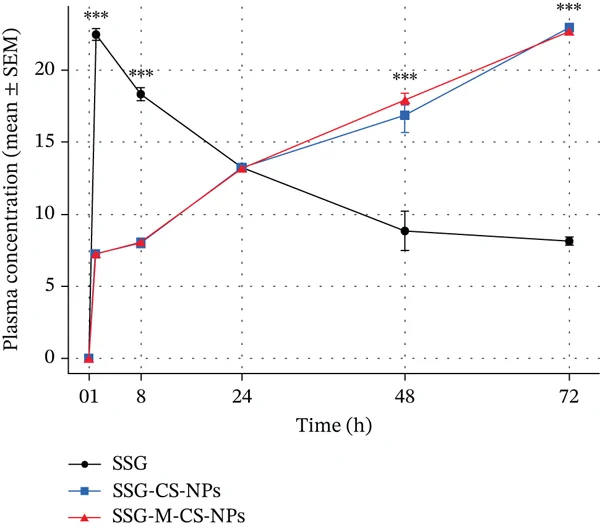
Pharmacokinetic profiles of free SSG and nanoformulations following administration in BALB/c mice. Plasma samples were collected at 0, 1, 8, 24, 48, and 72 h after treatment. Data are presented as mean ± SEM (*n* = 9/group). SSG, free sodium stibogluconate; SSG CS NPs, sodium stibogluconate chitosan nanoparticles; SSG M CS NPs, sodium stibogluconate chitosan mannose nanoparticles. Asterisks indicate a significant differences among the treatment groups at the indicated time points ( ^∗∗∗^ *p* < 0.001). No significant difference was detected among the groups at 24 h (*p* > 0.05).

In the free SSG group, the drug reached its peak plasma concentration 1 h after administration, reaching ~23 *μ*g/mL. Then, its plasma concentration began to decline, reaching 18.5 *μ*g/mL after 8 h and continued to decline until it reached its lowest concentration (~8 *μ*g/mL) after 72 h.

In the SSG NP groups (SSG CS NPs and SSG M CS NPs), a decrease in plasma drug concentration was observed after 1 h of treatment, reaching ~7.2 *μ*g/mL. However, the drug concentration continued to rise until it reached its peak after 72 h at ~23 *μ*g/mL.

### 3.2. Evaluation of Treatment Effectiveness

#### 3.2.1. ELISA

A one‐way ANOVA test revealed a statistically significant difference (*p* < 0.001), indicating significant variations in IgG levels among the study groups.

No significant differences were observed between the negative control group and the SSG, SSG CS NPs, and SSG M CS NPs treatment groups (*p* > 0.05). However, very low *p* values (*p* < 0.0001) indicated a highly significant difference between the treated groups (either free or nano SSG) compared with the positive control group. In addition, there were significant differences (*p* < 0.0001) between the SSG, SSG CS NPs, and SSG M CS NPs treatment groups and the dH_2_O‐treated group.

The results indicated that there were no statistically significant differences in the levels of IgG antibodies between the SSG, SSG CS NPs, and SSG M CS NPs groups (*p* > 0.05). Although there were no statistically significant differences, the mean of the SSG M CS NPs group (1.2) was slightly lower than that of the SSG and SSG CS NPs groups (2.1 and 1.81, respectively). (Figure 6).

**Figure 6:**
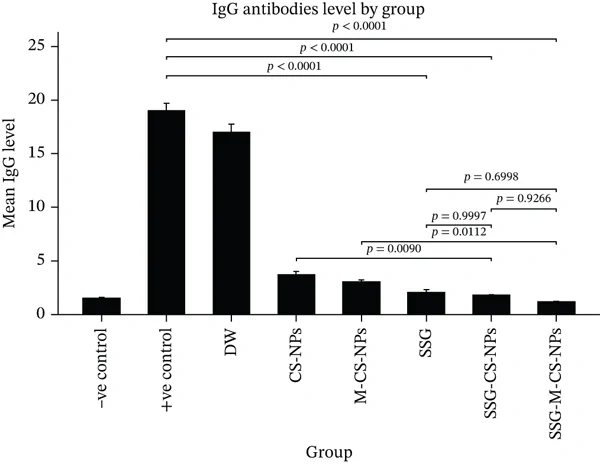
IgG antibody levels in the study groups measured 1 day after completion of treatment. Data are presented as mean ± SEM (*n* = 9/group). Statistical comparisons were performed using one‐way ANOVA followed by Tukey′s HSD post hoc test. Exact *p* values for the indicated pairwise comparisons are shown above the corresponding brackets; *p* < 0.05 was considered statistically significant. −ve control, negative control; +ve control, positive control; DW, infected mice administered distilled water; CS NPs, infected mice administered blank chitosan nanoparticles; M CS NPs, infected mice administered blank chitosan mannose nanoparticles; SSG, infected mice administered sodium stibogluconate; SSG CS NPs, infected mice administered with sodium stibogluconate chitosan nanoparticles; SSG M CS NPs, infected mice administered with sodium stibogluconate chitosan mannose nanoparticles.

Although the antibody levels were lower in the CS NPs and M CS NPs groups, statistically significant variations were seen between the SSG CS NPs and CS NPs groups (*p* = 0.009), as well as between the SSG M CS NPs and M CS NPs groups (*p* = 0.0112).

#### 3.2.2. Real‐Time qPCR Assay

The qPCR results for the SSG‐treated groups are summarized in Table 2. Three mice from each group were tested for qPCR positivity. One‐way ANOVA revealed a significant difference between these groups (*p* < 0.001).

**Table 2:** Quantification of parasitic load using qPCR across SSG experimental groups (*n* = 3).

Groups	Mean *C* *t* value ± S.E.M.	*Leishmania* detection result
−ve control	44.15847 ± 2.57	Negative
+ve control	31.8608 ± 0.79	Positive
DW	31.94347 ± 1	Positive
CS NPs	36.56433 ± 0.55	Positive
M CS NPs	38.4096 ± 0.63	Positive
SSG	39.05007 ± 1.54	Positive
SSG CS NPs	40.4146 ± 0.57	Negative
SSG M CS NPs	40.496 ± 2.21	Negative

The positive control group recorded the lowest *C* *t* values (31.8608), reflecting a high parasite load, whereas the negative control group showed the highest values (≥ 40), indicating the absence of the parasite.

The results also showed statistically significant differences between the positive control group and all treated groups (*p* < 0.05), with *C* *t* values increasing in the treated groups compared with the positive control, indicating a decrease in parasitic load.

Furthermore, the groups treated with the drug‐loaded nanoparticles (SGG CS NPs and SGG M CS NPs) showed higher *C* *t* values compared with blank nanoparticles (CS NPs and M CS NPs) with clear significant differences (*p* < 0.05), indicating improved drug efficacy after loading onto the nanoparticles. On the other hand, the SSG M CS NPs group exhibited the highest *C* *t* values among all treated groups, surpassing both the free SSG and the nonmannose‐coated formulation (SSG CS NPs). This difference was statistically significant when compared with the free SSG (*p* < 0.05), whereas no significant difference was observed when compared with SGG CS NPs (*p* > 0.05).

## 4. Discussion

The nanoparticle characterization results showed that the prepared particles, as shown in TEM images, had a regular shape and an average size of 32 ± 2 nm. This is consistent with previous studies demonstrating that the small size and regularity of nanoparticles facilitate endocytosis by macrophages and enhance drug‐targeting efficiency [44, 45].

The encapsulation efficiency of SSG within the nanoformulations reached 65.31%, indicating successful drug loading into the nanoparticles. This was attributed to the presence of hydrogen bonds and electrostatic charges between SSG and the carrier materials, such as chitosan or mannose‐conjugated chitosan. This finding aligns with the results of Esfandiari′s study [45], which indicated that such interactions contribute to improving the retention efficiency and stability of drug molecules within nanoparticles.

The FTIR spectroscopy results of the SSG‐loaded nanoformulation showed no significant changes in the positions of the characteristic peaks of the polymer compared with those of the unloaded polymer. This indicates that the addition of SSG did not affect the structural properties of the drug or the chemical stability of the nanoformulation. This confirms the stability of the chemical structure of the drug and prevents interactions that could impair its therapeutic efficacy [46].

Regarding the zeta potential evaluation, the positive zeta potential of the SSG CS NPs was 31.3 ± 0.97 and that of the SSG M CS NPs was 28.8 ± 0.55. These particles exhibited a stable positive charge. The relatively high positive values of surface potential generate electrostatic repulsion between particles, ensuring the electrical stability of the drug molecules and making precipitation or agglomeration unlikely to occur during storage or in vivo distribution [47, 48].

As for the in vitro release study of SSG, the results showed that after 350 h, more than 90% of the free SSG was released, compared with only 80% in the case of nanoformulations. This suggests that the nanoformulation of the drug has a longer release period, and thus, longer bioavailability, enhancing the therapeutic efficacy of SSG, as demonstrated by Riezk [17] in similar studies. This effect stems from the gelatinous and crosslinked nature of chitosan, which provides sustained drug release, contributing to enhanced therapeutic efficacy and reduced dosing frequency [49].

The study results showed that the nanoformulations of SSG (SSG CS NPs and SSG M CS NPs) maintained significantly higher and sustained drug levels in the plasma, reaching nearly three times those recorded for free SSG after 72 h. This indicates extended drug release and longer bioavailability compared with free SSG. This result is in agreement with the study conducted by Kip et al. [50], which indicated that SSG in its conventional form is rapidly eliminated by renal excretion within 2 to 3 h. Therefore, the bioavailability of free SSG in the body is short, necessitating repeated therapeutic doses and increasing the patient′s burden of receiving multiple treatment injections [51].

In contrast, with SSG nanoformulations, a longer drug release rate and bioavailability in the body were observed. This occurs because the nanoparticles encapsulate the drug and allow its sustained release into the plasma, improving therapeutic outcomes and reducing the patient′s drug dose [52].

These results are consistent with numerous studies indicating that the use of nanoformulations offers a significant advantage in improving drug pharmacokinetics, potentially contributing to increased treatment efficacy and reducing side effects associated with repeated or high doses of free drugs [53, 54]. This could also improve drug distribution in target tissues and the treatment outcomes for leishmaniasis [55, 56].

The controlled release of SSG in vitro effectively mirrored the drug′s in vivo behavior, demonstrating the consistency between the in vitro release behavior of SSG and its pharmacokinetics in plasma. This confirms the effectiveness of nanoparticles as drug delivery systems for regulating drug release and extending drug retention in the bloodstream.

ELISA results showed a significant decrease in IgG antibody levels in BALB/c mice infected with leishmaniasis and treated with SSG compared with the positive control group and the dH_2_O group, where the IgG antibody level was significantly higher. However, no statistically significant difference was observed between the SSG‐treated and negative control groups, which were not exposed to infection. These findings indicate that changes in IgG antibody levels are associated with treatment with SSG, both in its free form and when encapsulated in nanoparticles.

It was noted that the SSG M CS NPs group (loaded with mannose‐decorated CS NPs) showed the lowest mean IgG values among the treatment groups, followed by the SSG CS NPs group (loaded with CS NPs), indicating a significant biological improvement in treatment efficacy. However, the differences among the three treatment groups did not reach statistical significance. This is due to the unique properties of chitosan when incorporated into nanoparticle formulations, including its low toxicity and excellent drug delivery capacity because of its ability to easily cross cell membranes. Its high positive charge allows it to adhere to the cell membrane and easily enter negatively charged target cells, such as macrophages infected with *Leishmania* parasites [57, 58].

Moreover, the addition of mannose in the design of drug nanocarriers has greatly contributed to the targeted delivery of these drugs [59]. In the current study, the enhanced therapeutic efficacy observed with the SSG M CS NPs may be partially attributed to improved properties of the nanocarrier system, which may contribute to more efficient drug delivery to the site of infection. Although mannose functionalization has been reported to facilitate receptor‐mediated uptake in previous studies, direct evidence of mannose receptor targeting was not investigated in the present work [60]. In this context, lower IgG levels were observed in the SSG M CS NPs‐treated group compared with the positive control group, indicating a difference in the treatment‐associated serological response and supporting the potential benefit of this formulation within the parameters evaluated in the present study.

There was also a statistically significant difference between the SSG CS NPs group and the CS NPs group, as well as between the SSG M CS NPs group and the M CS NPs group. This indicates that the nanoloading of SSG drug inside chitosan particles or chitosan conjugated with mannose was more effective in delivering SSG to infection sites compared with blank particles. This represents an important step toward improving cellular uptake and reducing infection‐associated antibody levels, thereby enhancing therapeutic outcomes compared with conventional systems [45, 61].

Although IgG antibody levels provide supportive information during treatment evaluation, they should not be considered a direct indicator of parasite clearance. Therefore, in the present study, the IgG results were interpreted as one of the evaluated parameters and were not used as a standalone indicator of therapeutic efficacy.

Several previous studies have shown that the relationship between the *C* *t* value in the qPCR test and the amount of parasite DNA is inversely proportional; lower *C* *t* values indicate the presence of a greater amount of parasite DNA, indicating that the treatment was less effective, whereas higher *C* *t* values indicate a lower amount of DNA and, therefore, greater treatment effectiveness [62, 63].

In the present study, *C* *t* values were used for relative comparison of the amount of detectable parasite DNA among the experimental groups rather than for absolute quantification of parasite burden. Under the assay conditions used, higher *C* *t* values indicate relatively lower amounts of detectable parasite DNA. However, differences in *C* *t* values should be interpreted alongside the other parameters evaluated in the study and should not, on their own, be considered direct evidence of parasite clearance or therapeutic efficacy.

The qPCR data revealed very low *C* *t* values for the +ve control and distilled H_2_O groups, reflecting the presence of a high amount of parasite DNA, as expected in the absence of any effective treatment, relative to the negative cohort with a very high *C* *t* value indicating the absence of parasite DNA, consistent with its role as a negative control group.

The SSG‐treated groups exhibited higher *C* *t* values (SSG = 39.05007, SSG CS NPs = 40.4146, SSG M CS NPs = 40.496) than the positive control group (31.8608), indicating relatively lower levels of detectable parasite DNA (*p* < 0.05), with the magnitude of the effect varying among the treatment formulations.

Groups treated with SSG‐loaded nanoparticle formulations also exhibited higher *C* *t* values compared with the blank nanoparticle carriers, with statistically significant differences (*p* < 0.05). Under the experimental conditions, the higher *C* *t* values are consistent with lower detectable parasite DNA. These findings may be associated with the improved drug delivery properties of the nanoparticle formulations [64], as supported by the in vitro release profile and pharmacokinetics results which demonstrated sustained drug release.

Among the important findings of the present study, the mannose‐coated formulation (SSG M CS NPs) exhibited the highest mean *C* *t* value among the treated groups, suggesting a relative reduction in the amount of detectable parasite DNA under the experimental conditions employed. This observation may be associated with the presence of mannose, which has been reported to facilitate the interaction of nanoparticles with macrophages through mannose receptors, potentially enhancing drug delivery to target cells, as described in previous studies [65]. However, cellular targeting and receptor‐mediated uptake were not directly evaluated in the present study.

## 5. Conclusion

There are many drawbacks to traditional antiparasitic medications, including SSG, which is conventionally used to treat leishmaniasis. Therefore, integrating nanotechnology with these medicines represents a promising option. The nanoformulations developed in the present study exhibited favorable physicochemical characteristics and good drug‐loading efficiency, along with an extended in vitro release profile under the experimental conditions and a more prolonged pharmacokinetic profile compared with the free drug. Treatment with the SSG‐loaded nanoformulations was also associated with lower IgG levels and differences in the amount of detectable parasite DNA compared with free SSG. Nevertheless, the present study has several limitations, including the exclusive use of male mice; the lack of evaluation of cellular immune markers, such as IFN‐*γ* and IL‐12; the lack of direct assessment of macrophage uptake; the absence of lesion‐size measurements; the potential contribution of mannose modification to macrophage uptake; the clinical response of the lesions; and the use of a previously published analytical method for determining plasma antimony concentrations without independent bioanalytical validation in mouse plasma. These aspects should be considered in future studies to provide a more comprehensive evaluation of the developed formulations.

## Author Contributions

Z.A.A.: Conceptualization, data curation, formal analysis, investigation, methodology, validation, software, visualization, writing—original draft, and writing—review and editing. N.T.Z.: Conceptualization, investigation, methodology, validation, visualization, writing—review and editing, and supervision. A.H.H.: Conceptualization, investigation, methodology, validation, visualization, writing—review and editing, and supervision.

## Funding

This study is funded by the KAU Endowment (WAQF).

## Disclosure

All authors have read and agreed to the published version of the manuscript.

## Ethics Statement

This study was performed in accordance with the ARRIVE guidelines and approved by the Ethical Committee of The Faculty of Veterinary Medicine, Assiut University, Assiut, Egypt, according to the OIE standards for the use of animals in research (Reference No: 0354).

## Conflicts of Interest

The authors declare no conflicts of interest.

## Supporting Information

Additional supporting information can be found online in the Supporting Information section.

## Supporting information


**Supporting Information 1** Table S1: Use of nanotechnology to deliver SSG for the treatment of leishmaniasis in experimental studies.

**Supporting Information 2** Table S2: The raw data obtained from the pharmacokinetic analysis of sodium stibogluconate, measured using UV–Vis spectrophotometric methods at different time points across the experimental groups.

**Supporting Information 3** Table S3: Raw ELISA data for IgG antibody levels in the study groups.

**Supporting Information 4** Table S4. The raw cycle threshold (*C* *t*) values obtained from qPCR analysis for the evaluation of parasite burden in all experimental groups.

## Data Availability

The data that supports the findings of this study are available in the Supporting Information of this article.
