# Supplementary material for: Nano‐Based Sodium Stibogluconate for Treatment of Cutaneous Leishmaniasis Caused by Leishmania major in Male Mice
Source: J Parasitol Res. 2026 Oct 22;2026:5461643. doi: 10.1155/japr/5461643 (PMC13687934; doi:10.1155/japr/5461643)
Supplement: Supplementary file 1 [file JAPR-2026-5461643-s002.pdf]

Table S1. Use of nanotechnology to deliver SSG for the treatment of leishmaniasis in experimental studies.

Drug	Delivery systems	Routes	Model of study	Tested parasite	Efficacy	References
SSG	Niosome	NR	Mice	Visceral leishmaniasis	More effective than free SSG.	[20]
SSG	Niosome Liposome	IV	Mice	*L. donovani*	Improved the efficacy of SSG ten-fold compared to free SSG.	[64]
SSG	Tuftsin-bearing liposomes	IV	Hamsters BALB/c mice	*L. donovani*	Enhanced the effectiveness of SSG compared to free SSG.	[61]
SSG	Non-ionic vesicular dextran	IV	BALB/c mice	*L. donovani*	Contributed to a four-fold increase in SSG residence time compared to free drug and increased concentration and therapeutic efficacy in target organs.	[52]
SSG	Stearylamine-bearing liposomes	IV	BALB/c mice	*L. donovani*	Showed significant reduction in parasite load in the spleen and liver of mice treated with this formulation.	[65]
SSG	Phosphatidylcholine-stearylamine-bearing liposomes	IV	BALB/c mice	*L. donovani*	Achieved an 85% reduction in parasite burden in the bone marrow and 95–98% in the spleen and liver.	[47]
SSG	Cationic liposomes	IV	BALB/c mice	*L. donovani*	Showed a significant reduction in parasite load of over 90% compared to free drug.	[48]
SSG	Nano-deformable liposomes	TP	BALB/c mice	*L. tropica*	Demonstrated strong anti-leishmanial activity, manifested by healing of skin lesions and significantly reducing the parasite burden compared to free SSG.	[21]
SSG + KTZ	Nano-elastic liposomes	TP	BALB/c mice	*L. mexicana*	Increased the drug's permeability through the skin by 5-fold and reduced the parasite load by 35-fold compared to free drug.	[44]

NR, not reported; SSG, sodium stibogluconate; KTZ, ketoconazole; IV, Intravenous; TP, Topical.
